# An association of Aquaporin-4 with the immunoregulation of liver pathology in mice infected with *Schistosoma japonicum*

**DOI:** 10.1186/s13071-015-0650-7

**Published:** 2015-01-21

**Authors:** Weiwei Zhang, Jifeng Zhu, Xian Song, Zhipeng Xu, Xue Xue, Xiaojun Chen, Xiaowei Yang, Yong Li, Xiaoxiao Dong, Sha Zhou, Wei Li, Yingying Qian, Feng Liu, Chuan Su

**Affiliations:** Department of Pathogen Biology & Immunology, Jiangsu Key Laboratory of Pathogen Biology, Nanjing Medical University, 140 Hanzhong Road, Nanjing, Jiangsu 210029 China; Department of Pharmacology, Jiangsu Key Laboratory of Neurodegeneration, Nanjing Medical University, 140 Hanzhong Road, Nanjing, Jiangsu 210029 China; Department of Oncology, The First Affiliated Hospital of Nanjing Medical University, 300 Guangzhou Road, Nanjing, Jiangsu 210029 China

**Keywords:** Aquaporin-4, *Schistosoma japonicum*, Granuloma, Th1, Th2, Th17, Treg cells

## Abstract

**Background:**

Schistosomiasis is a chronic parasitic disease that affects approximately 200 million people. In Schistosomiasis japonica and mansoni, parasite eggs were trapped in host liver and stimulated the CD4^+^T cell responses to regulate the formation of the granulomas. Subsequently, excessive granulomatous response in some heavily, and/or repeatedly infected individuals could result in chronic liver fibrosis and circulatory impairment. Thus, elucidation of the mechanisms of these responses will not only provide more information to better understand the mechanisms of the immunoregulation in schistosomiasis, but also help to design new therapies to control granuloma-associated immunopathology. The role of aquaporin-4 (AQP4) in water transport has been extensively investigated in the central nervous system (CNS). Recently, studies have shown that AQP4 expresses in immune system and lack of AQP4 in mice results in significantly less CD4^+^CD25^+^ T regulatory cells (Treg cells) under physiological condition, one of the subpopulations of CD4^+^T cells which restrains immunopathology in hosts with schistosomiasis. However, little information exists regarding the contribution of AQP4 to the immune regulation in schistosome infection.

**Methods:**

The liver granulomatous response in *S. japonicum*-infected AQP4 knockout (KO) mice and its wild-type (WT) littermates were detected by staining liver sections with hematoxylin and eosin. The generation of various CD4^+^ T subsets, including Th1, Th2, Th17, and Treg cells were analyzed by flow cytometry. In addition, the levels of total IgG, IgG1, IgG2a in serum of infected mice were detected by ELISA assay.

**Results:**

Our results showed an enhanced granulomatous response with increased accumulation of eosinophils and macrophages around eggs in the liver of AQP4 KO mice with Schistosomiasis japonica. In addition, our study demonstrated enhanced Th2 but reduced Th1 and Treg cells generation in AQP4 KO mice with Schistosomiasis japonica, which may, at least partly, account for the enhancement of the liver granuloma formation.

**Conclusion:**

Our study for the first time provides evidences that AQP4 has an association with the immunoregulation of the liver granuloma formation, which may confer a new option for schistosomiasis treatment.

## Background

Schistosomiasis is one of the most prevalent parasitic diseases infecting more than 200 million people with an estimated 600 million at risk worldwide [1,2]. In schistosomiasis japonica and mansoni, the most severe damage to the host is the immunopathology of liver caused by the schistosome eggs. During infection, schistosome eggs are trapped in host liver and stimulate the granulomatous response. Subsequently, significant fibrosis and circulatory impairment can develop in a subset of individuals who suffer extensive or repeated infection and/or lack of treatment. Consequently, much of the symptomatology of schistosomiasis is attributed to the egg-induced granulomatous response in schistosomiasis japonica and mansoni [3-6].

Many factors are reported to be involved in regulating the immunopathogenesis of schistosomiasis. CD4^+^ T cell is one of the key players in the regulation of the liver granuloma formation by differentiation into different effector subsets including T helper (Th) 1, Th2, Th17 and T regulatory cells (Treg cells) [3,7-18]. Studies showed that Th2 and Th17 cells upregulate [9,11,14,18], but Th1 cells downregulate the hepatic granuloma formation in schistosomiasis [11,15]. Meanwhile, Treg cells also play an important suppressive role in immunopathology control [12,13,16]. Therefore, a deeper understanding of the mechanisms of these immune regulations is necessary for the better control of pathology in schistosomiasis.

Aquaporin-4 (AQP4), a member of AQPs, was originally cloned in 1994 from lung tissue [19]. Studies show that AQP4 is highly expressed in the CNS and regulates brain volume homeostasis, cerebrospinal fluid production, and contributes to the pathogenesis of brain edema [20-22]. Recently, AQP4 has been suggested to play a significant role in autoimmunity and neuroinflammation as the target antigen of the autoimmune responses [23-25]. Our previous study has demonstrated that AQP4 is also expressed on a range of immune cells including dendritic cells, macrophages, natural killer cells, B cells and T cells, suggesting its potential involvement in the modulation of immunological functions. In addition, AQP4-deficient mice had significantly less proportion and absolute number of Treg cells under physiological conditions, resulting from impaired generation of thymic-derived Treg cells [26]. Therefore, it raises the question of whether AQP4 plays a role in the immunoregulation in the host liver pathology after schistosome infection.

In this study, we showed an enhanced granulomatous response and remarkably increased Th2 but reduced Th1 and Treg cells generation in *S. japonicum*-infected AQP4 KO mice, which suggests a potential role for AQP4 in the immunoregulation in schistosomiasis.

## Methods

### Ethics statement

Animal experiments were performed in strict accordance with the Regulations for the Administration of Affairs Concerning Experimental Animals (1988.11.1), and all efforts were made to minimize suffering. All animal procedures were approved by the Institutional Animal Care and Use Committee (IACUC) of Nanjing Medical University for the use of laboratory animals (Permit Number: NJMU 11–0121).

### Mice, parasite and infection

AQP4 KO mice were generated as previously described and were kept under environmentally controlled conditions (ambient temperature, 22°C; humidity, 40%) on a 12-h light/dark cycle with free access to food and water [27]. Mice were identified by RT-PCR analysis of tail samples and Western blot analysis of the cerebral cortex.

*Oncomelania hupensis* harboring *S. japonicum* cercariae (Chinese mainland strain) were purchased from Nanjing municipal center for disease control and prevention (Jiangsu, China).

Female eight-week old AQP4 WT and KO mice were infected with 12 cercariae of *S.japonicum* through the abdominal skin. At week 0, 3, 5, 8 post-infection, four mice from each experimental group were randomly chosen from the infected and normal control groups and sacrificed for further study.

### Worm and egg burden examination in the liver

At 0, 3, 5, 8 weeks post *S. japonicum* infection, mice from each experimental group were sacrificed and perfused with saline containing heparin to recover the adult worms. Two grams of the liver were digested with 5%KOH at 37°C overnight, and the numbers of eggs were determined by microscopic examination.

### Histopathological analysis

Mice livers were fixed for 48 h in 10% buffered formalin and then embedded in paraffin. The sections were prepared and stained with hematoxylin and eosin (HE). For every granuloma containing a single egg, the area of the granulomas in 50 visual fields (ten sections for each mouse and five random microscope fields for each section) from each mouse was calculated by computer-assisted morphometric analysis under a microscope (magnification: 100×) as previously described (Olympus, Tokyo, Japan) [28]. Only granulomas appearing as circular in section were measured. Granuloma sizes are expressed as means of areas measured in μm^2^ ± SD. For every granuloma containing a single egg, neutrophils, eosinophils, lymphocytes and macrophages in each granuloma were determined by microscopic examination (magnification: 400×) as previously reported (Olympus) [29,30]. Quantitation of neutrophils, eosinophils, lymphocytes and macrophages were performed by determining the mean number of positive-stained cells over each granuloma, which were from ten sections for each mouse and five microscope fields for each section under a microscope (magnification: 100×).

### Separation of lymphocytes from spleens, lymph nodes and livers

Single cell suspensions of spleens or lymph nodes from schistosome-infected or control mice at week 0, 3, 5 and 8 post-infection were prepared in PBS containing 1% FBS by mincing the mouse spleen and mesenteric lymph nodes (Gibco, Grand Island, NY) and using centrifugation. Red blood cells were lysed using ACK lysis buffer.

Hepatic lymphocytes were prepared as described previously with some modifications [31,32]. In brief, for preparation of single cell suspension of hepatic lymphocytes, infected or control mouse livers were perfused via the portal vein with PBS. The excised liver was cut into small pieces and incubated in 10 ml of digestion buffer (collagenase IV/dispasemix, Invitrogen Life Technologies, Carlsbad, CA) for 30 min at 37°C. The digested liver tissue was then homogenized using a Medimachine with 50-μm Medicons (Becton Dickinson, San Jose, CA) according to the manufacturer’s instructions. The liver suspension was resuspended in 5 ml PBS and then placed on a lympholyte M (Cedarlane, Ontaric, Canada) overlay in a 1:1 ratio. Cells were spun at 2,200 rpm for 20 minutes, collected from PBS/Lympholyte M interface, washed and suspended in PBS.

### Cell culture

For *in vivo* investigation, single cell suspension of spleens, lymph nodes or livers from schistosome-infected or normal mice at week 0, 3, 5, 8 post-infection were cultured in complete RPMI 1640 medium (Gibco) containing 10% FBS, 2 mM pyruvate, 0.05 mM 2-mercaptoethanol, 2 mM L-glutamine, 100 U of penicillin/ml and 0.1 mg/ml streptomycin. Subsequently, 2 × 10^6^ cells were stimulated with 25 ng/ml PMA and 1 μg/ml ionomycin (Sigma-Aldrich) in complete RPMI 1640 medium in the presence of 0.66 μl/ml Golgistop (BD Biosciences PharMingen, San Diego, CA) for 6 h at 37°C in 5% CO_2_ [33-35]. Cells were collected for staining and FCM analysis.

For *in vitro* antigen stimulation assays, 1 × 10^6^ splenocytes /well were cultured in 24-well plates and pulsed with 20 μg/ml SEA or complete RPMI 1640 medium alone for 72 h at 37°C in 5% CO_2_. 66 hours later, splenocytes were stimulated with 25 ng/ml PMA and 1 μg/ml ionomycin (Sigma, St. Louis, MO) in the presence of Golgistop for 6 h. Cells were collected for staining and FCM analysis.

### Cell staining and FCM analysis

For intracellular IFN-γ ⁄ IL-4 ⁄ IL-17 staining and detection, 2 × 10^6^ splenocytes, lymphocytes, or liver cells from schistosome-infected or normal mice were surface stained with anti-CD3-APC mAbs (eBioscience, San Diego, CA) and anti-CD4-FITC mAbs for 30 minutes. Cells were washed, fixed and permeabilized with Cytofix/Cytoperm buffer (BD Pharmingen) for 40 minutes and then intracellularly stained with PE-conjugated anti-IFN-γ, anti-IL-4 or anti-IL-17 respectively (eBioscience) for 60 minutes. Cells were gated on the CD3^+^ population for analysis of Th1, Th2, or Th17 cells.

For detecting the proportion of CD4^+^ CD25^+^ Treg cells, intracellular Foxp3 staining was performed according to the manufacturer’s protocol of the Mouse Regulatory T Cell Staining Kit (eBioscience). Briefly, 2 × 10^6^ splenocytes, lymphocytes or liver cells from schistosome-infected or normal mice were surface-stained with anti-CD3-PerCP, anti-CD4-FITC and anti-CD25-APC for 30 minutes, followed by fixation and permeabilization with Cytofix/Cytoperm buffer (BD PharMingen) for 40 minutes and intracellular staining with anti-Foxp3-PE for 15 minutes. Cells were gated on the CD3^+^CD4^+^ population for analysis of Treg cells.

### SEA and SWA preparation

The *S. japonicum* adult worms were sonicated as previously described for harvesting the soluble fraction as the *S. japonicum* adult worms antigen (SWA) [36]. *S. japonicum* eggs were extracted from the livers of infected mice and enriched. The *S. japonicum* soluble egg antigens (SEA) were then prepared by harvesting the homogenized eggs as previously described [37]. The SEA and SWA concentrations were both determined by bicinchoninic acid (BCA) assay.

### Antibody detection with ELISA

The SWA and SEA specific IgG, IgG1, and IgG2a antibodies in mouse sera were determined by standard ELISA using the SWA and SEA as the coated antigen [36,37]. HRP-conjugated rat anti-mouse IgG (Calbiochem, Darmstadt, Germany), IgG1 and IgG2a monoclonal antibodies (mAbs) (BD Pharmingen) were used. In brief, ELISA plates (Titertek Immuno Assay-Plate, ICN Biomedicals Inc., Costa Mesa, CA) were coated with 0.1 mg/ml of SEA or SWA in 50 mM carbonate buffer (pH 9.6) and incubated overnight at 4°C. Plates were washed three times with PBS (pH 7.6) containing 0.05% Tween-20 (PBS-T) and blocked with 0.3% (w/v) bovine serum albumin (BSA) in PBS for 1 h at 37°C. The plates were further washed three times with PBS-T and then incubated with the sera diluted with 0.3% BSA (1:100) at 37°C for 1 h. The plates were washed four times with PBS-T, followed by incubation with HRP-conjugated rat anti-mouse IgG, IgG1 and IgG2a (1:1000) for 1 h at 37°C. The plates were then washed five times with PBS-T and developed with tetramethyl-benzidine (TMB) substrate (BD Pharmigen) for 30 min. The optical density (OD) of the color developed in the plate was read at 450 nm using a BioRad (Hercules, CA) ELISA reader.

### Statistics analysis

All data are expressed as mean ± SD. The statistical analysis was performed using SPSS software. ANOVA was used to demonstrate changes in expression at different time-points of *S.japonicum* infection. Statistical significance of the difference between AQP4 KO and WT groups at same time points were analyzed by two tailed Student's *t*-test and P < 0.05 was considered significant.

## Results

### *S. japonicum* infection results in an exacerbated liver granulomatous inflammation in AQP4 KO mice

**Figure 1:**
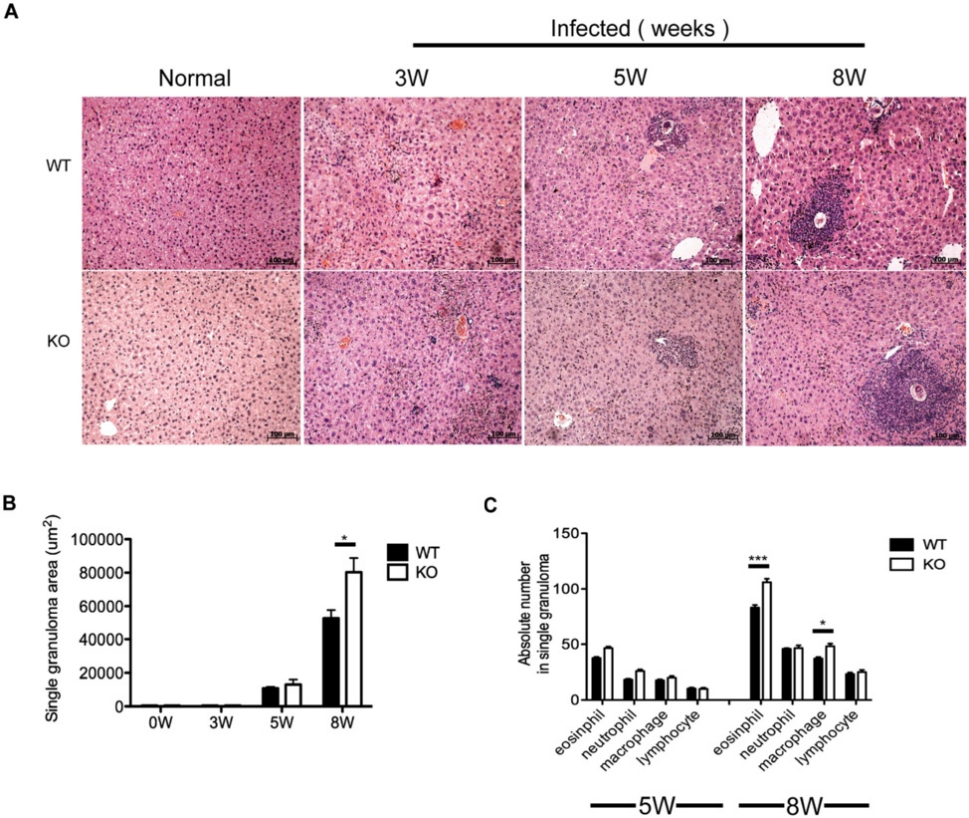
***S. japonicum*** **infection results in an exacerbated liver granulomatous inflammation in AQP4 KO mice.** At 0,3, 5,8 weeks post-infection, four AQP4 WT or KO mice were randomly chosen and sacrificed. Liver sections were stained with HE for microscopic examination. **(A)** Histopathology in the livers (magnification: 100×). Results are representative of two independent experiments. **(B)** Sizes of the granulomas were measured by computer-assisted morphometric analysis. **(C)** Absolute numbers of neutrophils, eosinophils, lymphocytes and macrophages in the granulomas. Values are given as mean ± SD of 8 AQP4 WT or KO mice from two independent experiments. *P < 0.05; **P < 0.01; ***P < 0.001.

Results showed that the granulomas developed after the deposition of parasite eggs in both AQP4 KO and WT mice livers. No later than 5 weeks post-infection, the average size of liver granuloma showed a quicker exacerbation in AQP4 KO mice and it was significantly larger than that in the WT mice 8 weeks post-infection (Figure 1A and B). In addition, the number of eosinophils and macrophages in granulomas in the liver of AQP4 KO mice was significantly increased, but there was no obvious difference in the number of lymphocytes and neutrophils between AQP4 KO and WT mice (Figure 1C). These data suggest that AQP4 may be involved in regulation of the granulomatous response after *S. japonicum* infection.

### Worm and egg burdens are similar in AQP4 KO and WT mice infected with *S. japonicum*

**Figure 2:**
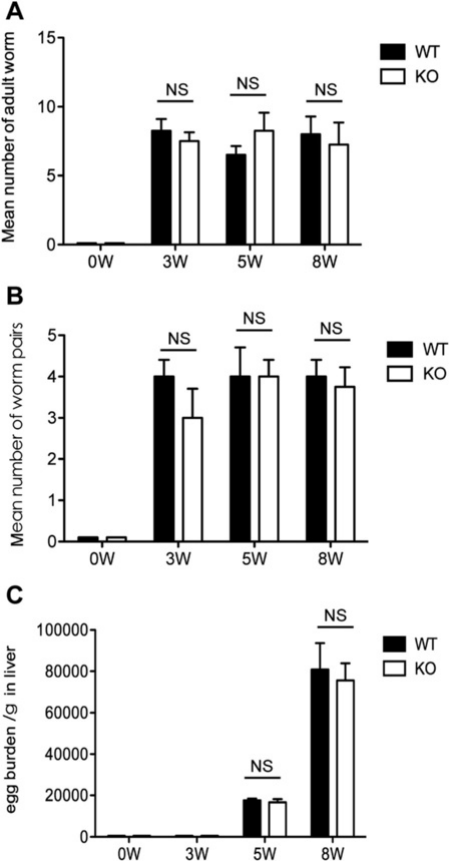
**Worm and egg burdens are similar in AQP4 KO and WT mice infected with** ***Schistosoma japonicum*** **.** At 3, 5, and 8 weeks after *S. japonicum* infection, four AQP4 WT or KO mice were randomly chosen and sacrificed and then perfused to calculate adult worms **(A)** or worm pairs **(B)**. **(C)** The number of eggs extracted from the liver was determined by microscopic examination. Values are given as mean ± SD of 8 mice from two independent experiments. *P < 0.05; **P < 0.01; ***P < 0.001.

The soluble egg antigen (SEA) secreted by matured schistosome miracidium within eggs is believed to cause a granulomatous response [38]. Results showed similar numbers of adult worms (Figure 2A), worm pairs (Figure 2B), and liver egg burden (Figure 2C) between AQP4 KO and WT mice. These results implicate that the enhanced granulomatous response in AQP4 KO mice with schistosomiasis japonica is caused by other mechanisms rather than difference in schistosome egg or worm burden.

### Th2 cell responses are stronger in *S. japonicum-*infected AQP4 KO mice

**Figure 3:**
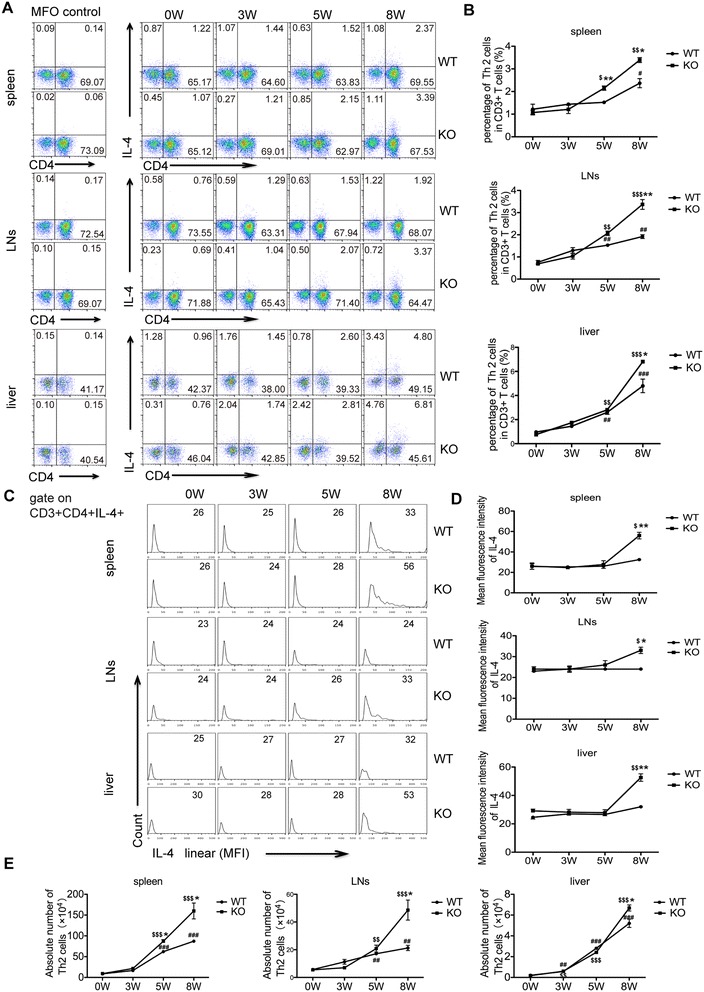
**Th2 cell responses are stronger in** ***S. japonicum-*** **infected AQP4 KO mice** ***.*** Four AQP4 WT or KO mice were randomly chosen and sacrificed at 0, 3, 5, 8 weeks post-infection. **(A)** FCM analysis of Th2 cell subsets in AQP4 WT and KO mouse splenocytes, mesenteric lymphocytes and hepatocytes. **(B)** The kinetics of the percentages (gated on CD3^+^ cells) of Th2 cells in total CD3^+^ T cells in AQP4 WT and KO mouse spleens, mesenteric lymph nodes and livers. Representative histograms obtained by FCM analysis **(C)** of mean fluorescence intensity (MFI) of IL-4 expression in Th2 cells **(D)**. **(E)** The kinetics of the absolute numbers of Th2 cells in AQP4 WT and KO mouse spleens, mesenteric lymph nodes and livers. Results are expressed as mean ± SD of 8 mice from two independent experiments. ^#^P < 0.05, ^##^P < 0.01, ^###^P < 0.001 vs. AQP4 WT-0 W; ^$^P < 0.05, ^$$^P < 0.01, ^$$$^P < 0.001 vs. AQP4 KO-0 W; *P < 0.05, **P < 0.01, ***P < 0.001 Th2 cells from AQP4 KO mice vs. from AQP4 WT mice at 0, 3, 5, 8 weeks post-infection.

It is widely accepted that schistosomiasis is associated with a Th2 – biased response caused by SEA, which is the key factor promoting the liver lesion [11,14]. As shown in Figure 3A and B, during the first 3 weeks post-infection the percentage of Th2 cells increased slowly in both AQP4 KO and WT mice and there was no apparent difference in Th2 responses between these two groups. Since week 5 post-infection, the proportion of Th2 cells in both AQP4 KO and WT mice increased markedly with a more rapid increase in the proportion of Th2 cells observed in AQP4 KO group. In addition, results in Figure 3C and D showed a higher mean fluorescence intensity (MFI) of IL-4 expression, which reflected the average level of IL-4 expressed in a single Th2 cell from AQP4 KO mice since 5 weeks post-infection. We further compared the absolute number of Th2 cells in spleens, mesenteric lymph nodes and livers of AQP4 KO and WT mice after infection. Consistently, more Th2 cells were present in AQP4 KO mice after 5 weeks post-infection (Figure 3E). These results suggest a correlation between the lack of AQP4 and higher Th2 cell responses during *S. japonicum* infection*.*

### Th17 cell responses show no statistically significant difference between AQP4 KO and WT mice after *S. japonicum* infection

**Figure 4:**
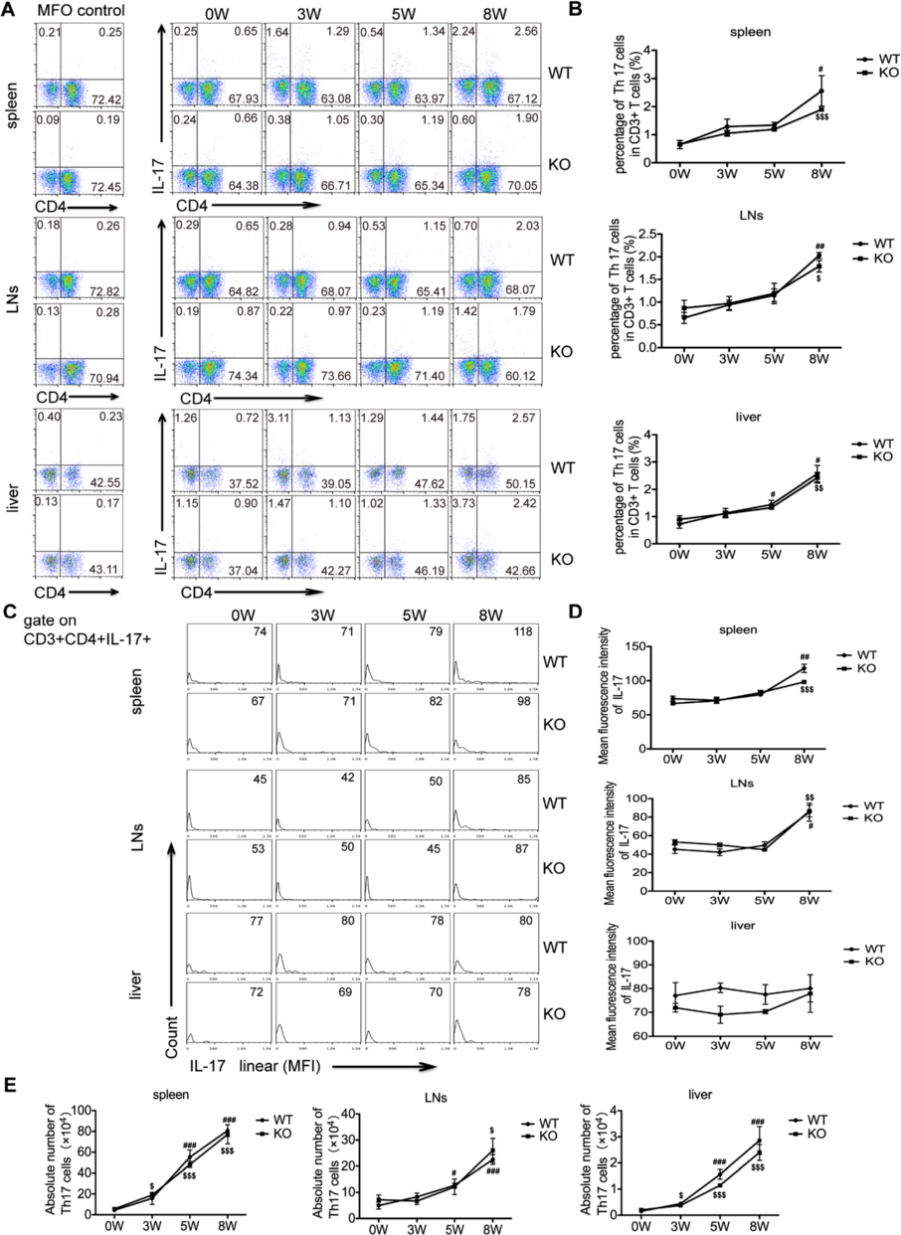
**Th17 cell responses show no statistically significant difference between AQP4 KO and WT mice after** ***S. japonicum*** **infection.** At 0, 3, 5, 8 weeks post-infection, four AQP4 WT or KO mice were sacrificed and single cell suspension of splenocytes, mesenteric lymphocytes or liver cells were prepared for FCM analysis of Th17 cells. **(A)** The cells were gated on CD3^+^ splenocytes,lymphocytes or liver cells from AQP4 WT or KO mice for the detection of Th17 cells. **(B)** The proportion (gated on CD3^+^ cells) of Th17 cells in the spleen, lymph nodes and livers. Representative histograms obtained by FCM analysis **(C)** of mean fluorescence intensity (MFI) of IL-17 expression in Th17 cell **(D)**. **(E)** The absolute number of Th17 cells in the spleen, lymph nodes and livers. Data represent means ± SD of 8 mice from two independent experiments. ^#^P < 0.05, ^##^P < 0.01, ^###^P < 0.001 vs. AQP4 WT-0 W; ^$^P < 0.05, ^$$^P < 0.01, ^$$$^P < 0.001 vs. AQP4 KO-0 W; *P < 0.05, **P < 0.01, ***P < 0.001 Th17 cells from AQP4 KO mice vs. from AQP4 WT mice at 0, 3, 5, 8 weeks post-infection.

Recent studies suggest that Th17 cells, which are primarily induced after egg deposition in host tissues, also promote the hepatic granuloma formation by secreting cytokine IL-17 [9,15,18]. The results in Figure 4 showed that the percentage and the absolute number of Th17 cells increased slowly during the first 3 weeks but increased quickly 5 weeks post-infection in both AQP4 KO and WT mice. However, there was no statistically significant difference in generation of Th17 cell between AQP4 KO and WT mice. The mean fluorescence intensity of IL-17 expression in Th17 cells showed no difference between AQP4 KO and WT mice at each stage of infection. These results indicate that AQP4 may not be involved in Th17 cell responses during *S. japonicum* infection.

### Th1 cell responses are decreased in *S. japonicum-*infected AQP4 KO mice

**Figure 5:**
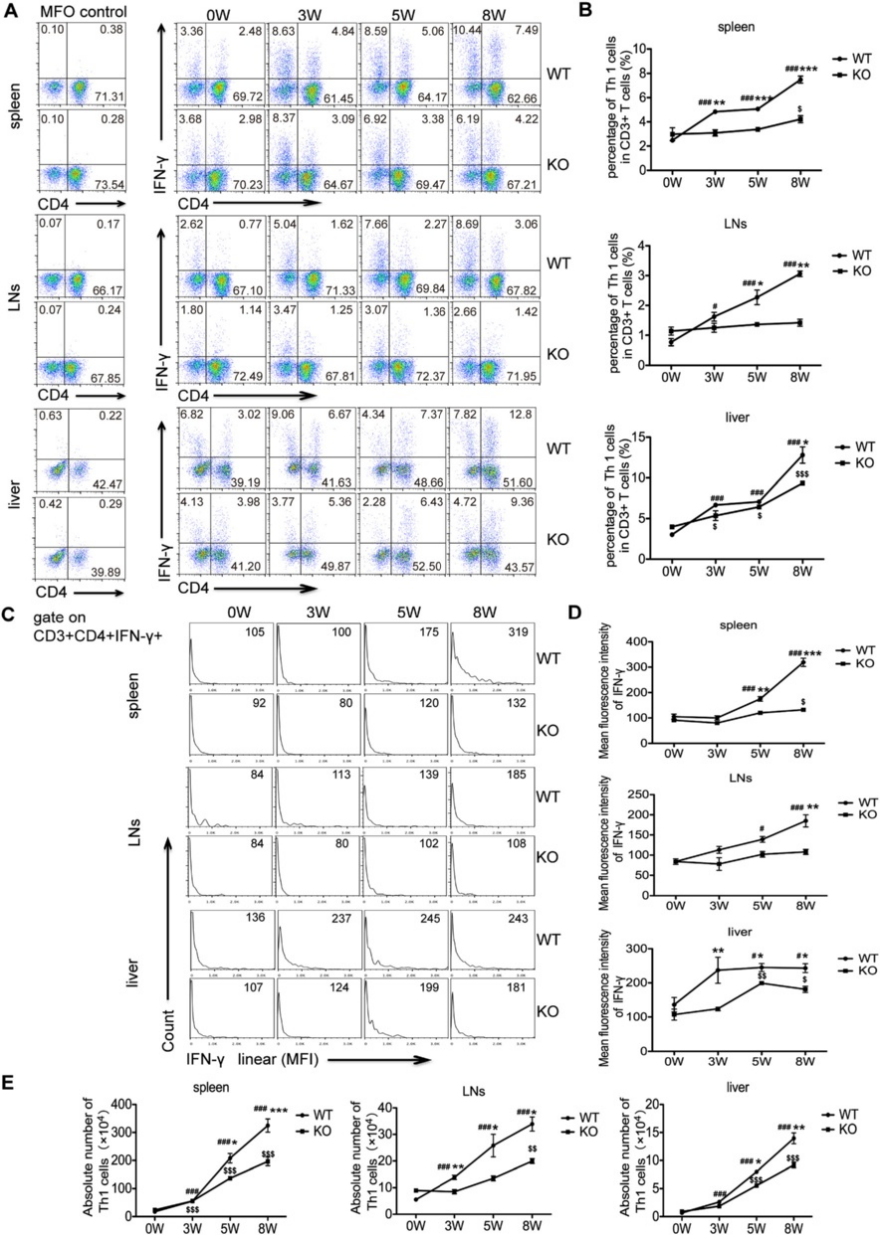
**Th1 cell responses are decreased in** ***S. japonicum-*** **infected AQP4 KO mice** ***.*** **(A)** At 0, 3, 5, 8 weeks post-infection, the generation of IFN-γ producing-CD3^+^CD4^+^ cells in the spleen, lymph nodes and liver of AQP4 WT and KO mice was determined by intracellular staining and FCM. **(B)** The proportion (gated on CD3^+^ cells) of Th1 cells in mouse spleen, lymph nodes and livers. Representative histograms obtained by FCM analysis **(C)** of mean fluorescence intensity (MFI) of IFN-γ expression in Th1 cells **(D)**. **(E)** The absolute number of Th1 cells in mouse spleen, lymph nodes and livers. Data represent means ± SD of 8 mice from two independent experiments. ^#^P < 0.05, ^##^P < 0.01, ^###^P < 0.001 vs. AQP4 WT-0 W; ^$^P < 0.05, ^$$^P < 0.01, ^$$$^P < 0.001 vs. AQP4 KO-0 W; *P < 0.05, **P < 0.01, ***P < 0.001 Th1 cells from AQP4 KO mice vs. from AQP4 WT mice at 0, 3, 5, 8 weeks post-infection.

An emergence of Th1 polarization is triggered after *S. japonicum* infection and is thought to down-regulate hepatic granuloma formation by secreting INF-γ in *S.japonicum* infection [11,15]. The results in Figure 5 showed that after 3 weeks post-infection, the increase in the percentage and the absolute number of Th1 cells in the spleen, lymph nodes, or liver of both AQP4 KO and WT mice was accelerated. However, Th1 cells in the AQP4 KO mice were notably less than those in WT control mice. In addition, the mean fluorescence intensity of IFN-γ expression was lower in Th1 cells from AQP4 KO mice 3 weeks post-infection. These results suggest a correlation between the lack of AQP4 and reduced generation of Th1 cells during *S. japonicum* infection.

### Treg cells are reduced in *S. japonicum-*infected AQP4 KO mice

**Figure 6:**
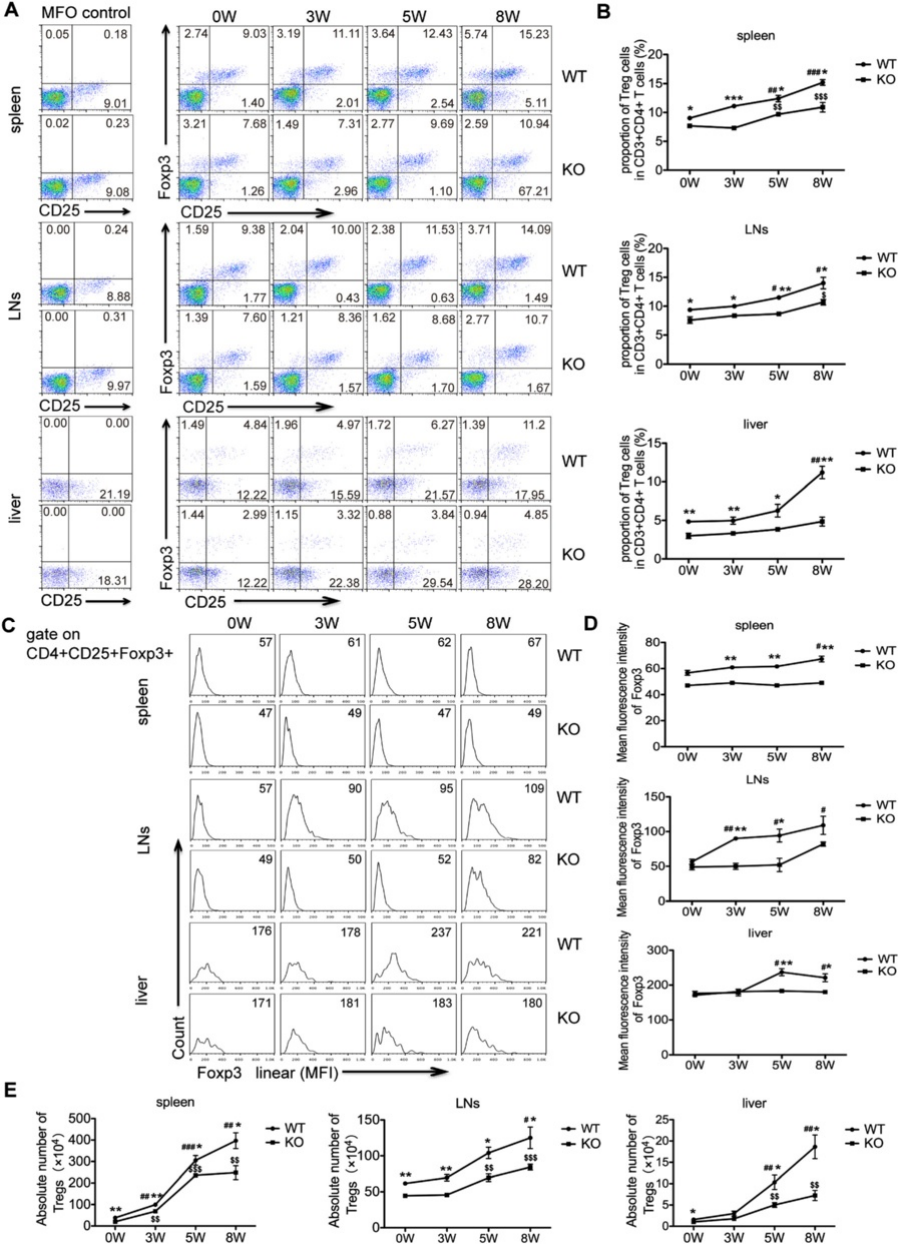
**Treg cells are reduced in** ***S. japonicum-*** **infected AQP4 KO mice** ***.*** **(A)** FCM analysis from one representative experiment. At 0, 3, 5, 8 weeks post-infection, four AQP4 WT or KO mice were sacrificed and single cell suspensions of splenocytes, mesenteric lymphocytes or liver cells were prepared for FCM analysis of Treg cells. **(B)** Proportions of Treg cells in CD3^+^CD4^+^ T cells isolated from the spleen, mesenteric lymph nodes, and liver. Representative histograms obtained by FCM analysis **(C)** of mean fluorescence intensity (MFI) of Foxp3 expression in Treg cells **(D)**. **(E)** The absolute number of Treg cells in the spleen, lymph nodes or liver from AQP4 WT and KO mice. Data represent means ± SD of 8 mice from two independent experiments. ^#^P < 0.05, ^##^P < 0.01, ^###^P < 0.001 vs. AQP4 WT-0 W; ^$^P < 0.05, ^$$^P < 0.01, ^$$$^P < 0.001 vs. AQP4 KO-0 W; *P < 0.05, **P < 0.01, ***P < 0.001 Treg cells from AQP4 KO mice vs. from AQP4 WT mice at 0, 3, 5, 8 weeks post-infection.

Studies have shown that CD4^+^CD25^+^Foxp3^+^ Treg cells are induced mainly by egg antigens during the infection, and play an important suppressive role in down-modulating granulomatous response in schistosomiasis [12,16]. Our results in Figure 6 showed that after *S. japonicum* infection, the proportion and the absolute number of Treg cells in AQP4 WT and KO mice were continuously increased. However, at each time point post-infection, the proportion and the absolute number of Treg cells in AQP4 KO mice were significantly less. Consistently, the mean fluorescence intensity of Foxp3 expression in Treg cells from AQP4 KO mice was less than that from AQP4 WT mice. These results suggest a correlation between the AQP4 deficiency and the reduction of Treg cells in mice during *S. japonicum* infection.

### CD4^+^ T cells from AQP4 KO mice display higher Th2 but lower Treg cells induction upon SEA stimulation *in vitro*

**Figure 7:**
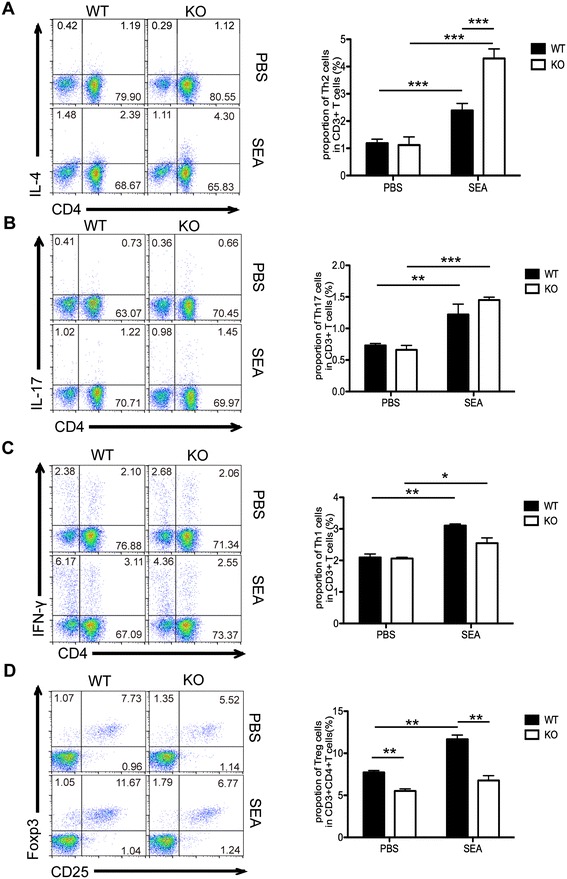
**CD4** ^**+**^ **T cells from AQP4 KO mice display higher Th2 but lower Treg cells induction upon SEA stimulation** ***in vitro*** **.** 8 weeks older AQP4 WT or KO mice were sacrificed, and single cell suspensions of splenocytes were prepared and *in vitro* stimulated with SEA as described in Materials and Methods for FCM. Cells were gated on the CD3^+^ population for analysis of proportions of Th2 **(A)**, Th17 **(B)**, and Th1 **(C)** cells in CD3^+^ T cells or on CD3^+^CD4^+^ population for analysis of proportion of Treg cells **(D)** in CD3^+^CD4^+^ T cells. FCM analyses were from one representative experiment. Results are expressed as mean ± SD of 24 mice from 3 independent experiments. *P < 0.05; **P < 0.01; ***P < 0.001.

As shown in Figure 7, in PBS control group, the proportion of Th2, Th17 and Th1 cells in AQP4 KO mice was similar to that in WT groups, while the Treg cells were significantly less in CD4^+^ T cells from AQP4 KO mice, indicating that AQP4 may regulate Treg cells at the steady state. Compared to the PBS control groups, SEA *in vitro* stimulation significantly promoted the proportions of Th1, Th2 and Th17 cells but only slightly increased Tregs in both AQP4 KO and WT mice. However, compared to AQP4 WT group, the differentiation of Th2 cells increased but the differentiation of Treg cells reduced from AQP4 KO group upon SEA *in vitro* stimulation. These results indicate that AQP4 deficiency leads to higher Th2 but lower Treg cells induction upon *in vitro* SEA stimulation.

### AQP4 KO mice show higher IgG1 but lower IgG2a levels after *S. japonicum* infection

**Figure 8:**
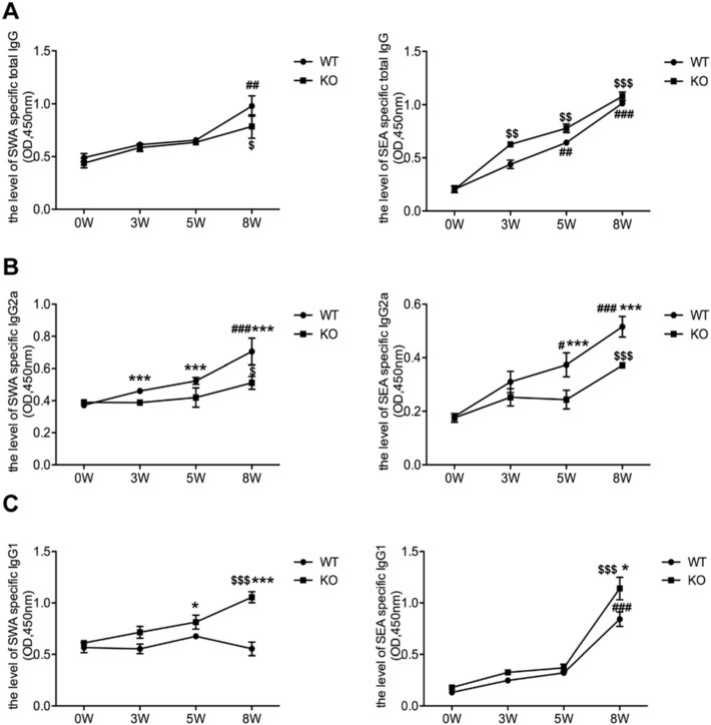
**AQP4 KO mice show higher IgG1 but lower IgG2a levels after** ***S. japonicum*** **infection** ***.*** At 0, 3, 5, 8 weeks post-infection, four AQP4 WT or KO mice were sacrificed and the serum samples were collected for standard ELISA using the SWA and SEA as the coated antigen. **(A)** The kinetics of the level of total IgG in the serum from AQP4 WT or KO mouse. SEA and SWA specific IgG2a **(B)** and IgG1 **(C)** antibodies in serum from *S. japonicum* infected AQP4 WT and KO mice were detected by ELISA. Results are expressed as mean ± SD of 8 mice from two independent experiments. ^#^P < 0.05, ^##^P < 0.01, ^###^P < 0.001 vs. AQP4 WT-0 W; ^$^P < 0.05, ^$$^P < 0.01, ^$$$^P < 0.001 vs. AQP4 KO-0 W; *P < 0.05, **P < 0.01, ***P < 0.001 total IgG, IgG1 and IgG2a cells from AQP4 KO mice vs. from AQP4 WT mice at 0, 3, 5, 8 weeks post-infection.

During schistosomiasis infection, IgG2a and IgG1 immunoglobulin isotypes are related to Th1 and Th2 cell responses, respectively [39]. The results in Figure 8 showed that after *S. japonicum* infection, the levels of total IgG and its subtypes IgG1 and IgG2a were increased in both AQP4 KO and WT mice. The levels of total IgG in AQP4 KO and WT mice displayed no significant difference (Figure 8A). However, at 3 weeks post-infection, the level of IgG2a in AQP4 KO mice was significantly lower than that in WT mice (Figure 8B), while at 5 weeks post-infection, a markedly higher level of IgG1 was observed in AQP4 KO mice compared with that in WT mice (Figure 8C). These results indicate AQP4 deficiency leads to the lower IgG2a but higher IgG1 levels in a *S. japonicum* infected mice*.*

## Discussion

Aquaporins (AQPs) were identified as a family of water channel proteins that provide a pathway for driving water transport through cell membranes for which the 2003 Nobel Prize in Chemistry was awarded to Peter Agre [40]. As a member of AQPs, AQP4 also has been known to contribute to regulate water homeostasis, especially in the CNS [20-22]. In our previous study, we reported that AQP4 is also expressed by various immune cells and lack of AQP4 was associated with reduced Treg cells under physiological conditions, suggesting a potential involvement of AQP4 in the immune regulation [26]. In this study, we showed that AQP4 deficiency leads to an increase in differentiation of Th2 cells but a decrease in differentiation of both Th1 and Treg cells during *S. japonicum* infection, and for the first time suggested a possible role of AQP4 in the immunoregulation of the liver pathogenesis in schistosomiasis.

In schistosomiasis japonica and mansoni, the egg-induced granulomatous response in the liver may eventually cause extensive fibrosis and development of portal hypertension in a subset of seriously and/or repeatedly infected individuals [4,8]. Therefore, elucidating the mechanisms that regulate the severity of schistosomiasis has been a major research objective. It is widely accepted that the liver granuloma formation is orchestrated by multiple subpopulations of CD4^+^ T cells including Th1, Th2, Th17, and Treg cells induced by schistosome egg antigens [13-15]. Our study showed that the granulomatous pathology and eosinophil infiltration were much more severe in AQP4 KO mice, which was consistent with an enhanced Th2 cells generation and the reduced Th1 and Treg cells generation in *S. japonicum-*infected mice AQP4 KO. Thus, it suggests not only an important role of AQP4 in CD4^+^T differentiation, but also a possible contribution of AQP4 to the immunoregulation of the granuloma formation in *S. japonicum-*infected host.

Our result did not show any differences in schistosome egg or worm burden between AQP4 KO and WT mice. This data is supported by the observation that no differences in Th1 response were observed before 3 weeks post-infection, the period of which is critical for host immune responses to kill the migrating schistosomulum. Thus, we speculate that although lack of AQP4 may play an important role in CD4^+^ T cell differentiation and the regulation of the granuloma formation, it may not be sufficient and/or necessary for the host’s early protective immunity against worm clearance or egg production.

Although it was evident that AQP4 may involve in CD4^+^ T cells differentiation by decreasing Th2 cells but increasing Th1 cells and Treg cells generation during *S. japonicum* infection, the underlying mechanism is interesting but not fully addressed in this study. It was demonstrated that deletion of AQP3 in dendritic cells could reduce the frequency of CD4^+^ cDCs and impair LPS-induced decrease of CD103^+^ dermal DCs, although the mechanism still remains unknown, which suggested AQP3 expressed on DCs regulate the development of DCs [41]. Thus, it is worth noting that AQP4 expression in CD4^+^ T cells or other immune cells may be directly involved in modulating CD4^+^ T cells differentiation pathways and the mechanism awaits further investigation. Furthermore, we can’t exclude that AQP4 deficiency may also have an effect via a very indirect mechanism. As AQP4 is expressed in the nervous system, it is possible, for example, that its absence might have an effect via neuroimmunological links, or, the mechanism perhaps involves both the immune system and the other system such as the nervous system. Thus, it may be preferential to develop AQP4 conditional knockout mouse models and significant research should be made in the future concerning mechanism how AQP4 regulate the polarization of Th cells and their actions to hepatic lesion.

## Conclusions

In summary, by using AQP4 KO mouse model of schistosomiasis japonica, we demonstrated for the first time an association of AQP4 with the immunoregulation of the liver pathology suggested an important role for AQP4 in regulation of CD4^+^ T cells differentiation in schistosomiasis. In addition, these novel findings imply that AQP4 may function as a new therapeutic target if it is directly involved in Th polarization pathways within immune system cells by modulating CD4^+^ T cell responses for schistosomiasis or other immune-associated diseases.

## Abbreviations

AQP4: Aquaporin 4
*S. japonicum*: *Schistosoma japonicum*
SWA: Schistosome worm antigen
SEA: Soluble egg antigen
Th1: T helper 1
MFI: Mean fluorescence intensity
FCM: Flow cytometry
